# Low *BRAF* and *NRAS* expression levels are associated with clinical benefit from DTIC therapy and prognosis in metastatic melanoma

**DOI:** 10.1007/s10585-013-9587-4

**Published:** 2013-05-15

**Authors:** Einar Birkeland, Christian Busch, Elisabet Ognedal Berge, Jürgen Geisler, Göran Jönsson, Johan Richard Lillehaug, Stian Knappskog, Per Eystein Lønning

**Affiliations:** 1 Section of Oncology, Institute of Medicine, University of Bergen, Bergen, Norway; 2 Department of Oncology, Haukeland University Hospital, Jonas Lies vei 26, 5021 Bergen, Norway; 3 Department of Oncology, Clinical Sciences, Lund University, Lund, Sweden; 4 CREATE Health Strategic Center for Translational Cancer, Lund University, Lund, Sweden; 5 Department of Molecular Biology, University of Bergen, Bergen, Norway; 6 Present Address: Teres Medical Group, Bergen, Norway; 7 Present Address: Institute of Medicine, University of Oslo, Oslo, Norway; 8 Faculty Division, Akershus University Hospital, 1478 Lørenskog, Norway

**Keywords:** Melanoma, BRAF, NRAS, Chemoresistance, Dacarbazine

## Abstract

Metastatic melanoma is characterized by a poor response to chemotherapy. Furthermore, there is a lack of established predictive and prognostic markers. In this single institution study, we correlated mutation status and expression levels of *BRAF* and *NRAS* to dacarbazine (DTIC) treatment response as well as progression-free and overall survival in a cohort of 85 patients diagnosed with advanced melanoma. Neither *BRAF* nor *NRAS* mutation status correlated to treatment response. However, patients with tumors harboring *NRAS* mutations had a shorter overall survival (*p* < 0.001) compared to patients with tumors wild-type for *NRAS*. Patients having a clinical benefit (objective response or stable disease at 3 months) on DTIC therapy had lower *BRAF* and *NRAS* expression levels compared to patients progressing on therapy (*p* = 0.037 and 0.003, respectively). For *BRAF* expression, this association was stronger among patients with tumors wild-type for *BRAF* (*p* = 0.005). Further, low *BRAF* as well as *NRAS* expression levels were associated with a longer progression-free survival in the total population (*p* = 0.004 and <0.001, respectively). Contrasting low *NRAS* expression levels, which were associated with improved overall survival in the total population (*p* = 0.01), low *BRAF* levels were associated with improved overall survival only among patients with tumors wild-type for *BRAF* (*p* = 0.013). These findings indicate that *BRAF* and *NRAS* expression levels may influence responses to DTIC as well as prognosis in patients with advanced melanoma.

**Electronic supplementary material:**

The online version of this article (doi:10.1007/s10585-013-9587-4) contains supplementary material, which is available to authorized users.

## Introduction

The incidence of cutaneous malignant melanoma is increasing among light-skin Caucasians [1]. Although most patients are cured by surgical excision of the tumor, approximately 20 % will relapse [2]. Systemic treatment for metastatic malignant melanomas remains palliative. Dacarbazine (DTIC) is considered standard chemotherapy treatment, even though objective response rates as low as 10 % are recorded [3].

Activating mutations in the *BRAF* (V600E) and *NRAS* (Q61K) genes are found at a frequency of 40–60 and 15–30 % in metastatic melanomas, respectively [4–10]. In vitro studies have shown that the V600E mutation, which is located in the protein’s activation loop, causes a 500-fold increase in the enzymatic activity of BRAF, enhancing activation of its downstream target, ERK [11]. Thus, for tumors harboring *NRAS* or *BRAF* mutations, activation of this pathway is thought to play a key role in driving tumor growth. This is further underlined by recent studies showing that targeted inhibition of mutated BRAF may cause tumor regression in metastatic melanomas harboring BRAF V600E mutations [12, 13], as well as findings indicating that treatment of *BRAF*-mutated melanomas may benefit from inhibition of the downstream effector MEK [14].

While the effects of the V600E mutation of *BRAF* have been extensively studied in experimental systems, several aspects of *BRAF* function remain poorly understood. Interestingly, copy-number gains of the *BRAF* gene have been proposed as an alternative mechanism of activation in both melanoma and glioma [15, 16], as well as being a cause of resistance towards BRAF inhibitor treatment of advanced melanoma [17]. Further, *BRAF* mRNA has been found subject to alternative splicing, with different transcript variants identified in colorectal cancer as well as in melanoma [18, 19]. Interestingly, expression of some splice variants has been related to resistance toward the BRAF inhibitor vemurafenib [19].

Although overexpression of wild-type *BRAF* has been reported to be an underlying mechanism of pathway activation in experimental systems [15], to the best of our knowledge the level of *BRAF* expression in tumors wild-type for *BRAF* has not been investigated as a potential predictive and prognostic factor in melanoma patients.

The aim of this study was to evaluate the predictive and prognostic impact of genetic disturbances and expression levels of *BRAF* together with *NRAS* mutations in patients treated with DTIC monotherapy for advanced melanomas.

## Materials and methods

### Patients and treatment

A total of 85 patients were enrolled in this protocol between January 2000 and November 2007. All patients were referred to the Department of Oncology at Haukeland University Hospital for locally advanced or metastatic melanoma. The protocol was approved by the Regional Ethical Committee, and was conducted in adherence to the Declaration of Helsinki. Each patient signed a written consent form. Details regarding the patient population studied are described in Table 1 and Supplementary Information, Table S1. Chemotherapy consisted of DTIC monotherapy, administered at a dose of 800–1,000 mg/m^2^ on a 3-weekly basis. Out of the total number of 85 patients, 75 commenced on chemotherapy and were available for response evaluation (the reason for non-compliance from the additional 10 patients is shown in Table S1). Evaluation of response was done at 6-weekly intervals. As the protocol was implemented in year 2000, the UICC response criteria were used for the whole series.

**Table 1:** Patient characteristics according to *BRAF* and *NRAS* mutation status

	Total patient population	*BRAF*		*NRAS*	
		WT	Mutant	WT	Mutant
Patient characteristics					
Median age (range)	62 (25–86)	62 (38–84)	63 (25–86)	63 (25–86)	61 (43–80)
Male (%)	56.5	60	50	57.4	52.9
Previous primary melanoma					
Cutaneous	57^a^	34	22	44	12
Breslow thickness					
<1	6	3	3	6	0
1–2	15	6	9	12	3
2–4	11	10	1	8	3
>4	16	12	4	11	5
Not available	8	3	5	7	1
Acral	4	4	0	4	0
Mucosal	5	4	1	5	0
Uveal	5^a^	5	0	4	1
No primary	15	8	7	11	4
Serum LDH (% elevated)	39.7	47.2	24.0	36.1	52.9
Metastases at inclusion^b^					
Soft tissue^c^	20	12	8	17	3
Visceral/skeletal	54	33	21	44	10
Brain	11	10	1	7	4
Stage at inclusion					
III	3	1	2	3	0
IV	82	54	28	65	17
Clinical benefit (%)	20	20.8	18.5	20.3	18.8

^a^One patient presented with both uveal and cutaneous primary

^b^Refers to the metastatic site associated with the worst prognosis

^c^Defined here as subcutaneous, cutaneous or lymph node metastases

### Tumor tissue collection and handling

Prior to chemotherapy, tumor samples were obtained through incisional biopsies or ultrasound-guided tru-cut needle samples from deep lesions (liver deposits). Tissue samples were snap-frozen in the operating theatre immediately upon removal and stored in liquid nitrogen until analysis. In addition, some of the excised material was formalin-fixed and paraffin-embedded for histological examination.

### Isolation of nucleic acids and cDNA synthesis

Total RNA was extracted using Trizol^®^ reagent (Invitrogen, Carlsbad, CA, USA) according to the manufacturer’s instructions. Some of the samples contained high levels of melanin after the RNA extraction; here, further steps were performed to separate RNA from melanin [20]. Following purification, RNA was dissolved in DEPC-treated dH_2_O and stored at −80 °C.

DNA was extracted from the biopsies using QIAamp DNA Mini Kit^®^ (Qiagen, Chatsworth, CA, USA). Some of the DNA samples also had to undergo further steps to separate DNA from melanin [21, 22]. DNA was dissolved in dH_2_O and kept at −20 °C.

First strand complementary DNA (cDNA) was synthesized using the Transcriptor reverse transcriptase (Roche Diagnostics, Basel, Switzerland) following the manufacturer’s instructions, using oligo-dT as primer and 250 ng total RNA as template.

### *BRAF* and *NRAS* mutation screening

Regions covering the open reading frame of *NRAS* and the 3′ half of the *BRAF* open reading frame was amplified from cDNA by PCR. In about half of the cases (*n* = 47), findings in *BRAF* were subjected to confirmatory analyses performed on genomic DNA. The PCR amplifications were performed using the DyNazyme EXT polymerase system (FINNZYMES, Espoo, Finland) according to the manufacturer’s instructions. Primers and annealing temperatures used are listed in Supplementary Information, Table S2. DNA sequencing was performed using BigDye v.1.1 and a capillary DNA sequencer (ABI 3700).

### Quantification of *BRAF* and *NRAS* mRNA transcripts

The expression levels of *BRAF* and *NRAS* and the two splice variants BRAFdel14–15 and BRAFdel12–15 were assessed by qPCR using the LightCycler 480 system (Roche Diagnostics, Basel, Switzerland). Specific amplification of each splice variant was ensured by using unique forward primers encompassing the specific splice site in combination with hybridization probes and reverse primers that were targeted to shared regions (primers and probes are listed in Supplementary Information, Table S2). To determine the specificity of the reactions, all reaction conditions were tested against a purified full-length template as well as other non-specific splice variants. The relative non-specific amplification of *BRAF* full-length/splice variants was determined to be lower than 5 × 10^−4^ as compared to specific amplification in all assays. Expression levels were normalized to ribosomal protein, large, P2 (RPLP2) expression before relative comparison between samples. Three patients were excluded from correlational analyses of *BRAF* full-length and *NRAS*, and an additional seven from splice variant mRNA analyses, due to total RNA levels falling below the sensitivity threshold of each assay.

### Statistical analysis

Due to the low rate of objective response (*n* = 4), analysis of potential predictive factors was carried out by categorizing patients as having either a clinical benefit (Complete response, CR; partial response, PR; and stable disease, SD; *n* = 15), or progressive disease (PD; *n* = 60), as previously described for this patient cohort [23]. Correlating *BRAF* and *NRAS* expression levels to therapy response, we compared relative expression levels among responders versus non-responders using the Mann–Whitney rank test. Relative expression levels among *BRAF* mutation and amplification carriers were compared using the Mann–Whitney and Kruskal–Wallis rank tests. For correlation analyses, Spearman’s rank correlation coefficients were calculated. Comparisons of categorical data were performed using Fischer’s exact test. Assessing the prognostic role of *BRAF* and *NRAS* mRNA expression levels, we used the median value as cut-off limit. Log rank tests and Cox regression analyses were used for survival analyses. All *p* values are given as two-sided, and the *p* values from Fischer exact tests present cumulative values. All analyses were performed using the SPSS 19.0.0.2 statistical software package (Chicago, Illinois, USA).

## Results

### *BRAF* and *NRAS* mutation status

The *BRAF* single nucleotide substitution c.1799T>A (p.V600E) was detected in 29, and c.1798_1799GT>AA (p.V600K) in 1 out of 85 patients (35.3 % harboring *BRAF* mutations in total). No other mutations were observed in the region covered by sequencing (codon 463–715).

Sequencing of the protein-coding region of the *NRAS* gene revealed mutations in 19 out of 85 (22.4 %) patients, all located in the codon 61 hot spot. Notably, *BRAF* and *NRAS* mutations were found to be mutually exclusive (*p* < 0.001, Supplementary Information, Table S1), with no tumor harboring mutations in both genes. A summary of patient characteristics in regards to *BRAF* and *NRAS* mutation status is presented in Table 1.

Interestingly, the incidence of *BRAF*, but not *NRAS* mutations varied according to the anatomical site from which the sample was derived. Thus, *BRAF* mutations were found in 15/42 (35.7 %) of subcutaneous metastases, 12/20 (60.0 %) of lymph node metastases, but only in 3/22 (13.6 %) of the visceral metastases examined (*p* = 0.007; details available in Supplementary Information, Table S1). Studies have shown that uveal and mucosal primary tumors only rarely carry mutations in *BRAF* and *NRAS* [24]. Excluding metastatic deposits with primary tumors at these sites, which represented a substantial fraction of the visceral metastases (*n* = 9), from the analysis, moderated the difference observed (leaving 3/13 visceral deposits from cutaneous melanomas harboring *BRAF* mutations; 23.1 %), still, a significant difference in mutation distribution between metastases located to different organ systems remained (*p* = 0.040).

In a previous paper [25] using CGH array, we reported *BRAF* copy-number gains in 12 out of a subgroup of 53 samples of the tumors studied here. Interestingly, 9 out of 12 tumors with increased *BRAF* copy number were found to harbor *BRAF* V600 mutations in concert. In contrast, *BRAF* mutations were recorded in 16 out of 41 melanomas without *BRAF* gains, revealing a statistically significant association between *BRAF* copy-number gains and V600 mutation status (*p* = 0.047).

### *BRAF* mRNA expression levels are elevated in metastatic melanoma and are higher in tumors harboring *BRAF* copy-number gains or V600 mutations

**Fig. 1:**
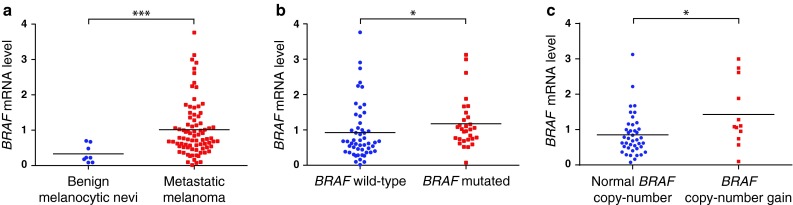
Expression characteristics of *BRAF* mRNA. Expression levels of *BRAF* mRNA in: **a** benign melanocytic nevi and in metastatic melanoma biopsies, **b** tumors with wild-type or mutated *BRAF*, and **c** tumors with or without *BRAF* copy-number gains. *Asterisks* denote significance levels: *p ≤ 0.05, **p ≤ 0.005, ***p ≤ 0.001

Expression levels of full-length *BRAF* mRNA was significantly higher in the malignant melanoma samples as compared to non-malignant nevi (*p* = 0.007, Fig. 1a).

While analyzing for *BRAF* mutations, we observed two alternatively spliced variants, one lacking exons 14 and 15 (BRAFdel14–15); and one lacking exons 12–15 (BRAFdel12–15). Of these, the former has previously been reported in colorectal cancer [18], whereas the latter is novel.

We determined the mRNA expression levels of *BRAF* full-length and the two alternatively spliced variants of *BRAF* by qPCR. The expression levels of the alternatively spliced variants, BRAFdel14–15 and -12–15, were lower than the full-length transcript, and the expression levels of each correlated positively with the expression level of full-length *BRAF* (*r*_s_ = 0.6609; *p* < 0.001 and *r*_s_ = 0.4604; *p* < 0.001, respectively; Supplementary Information, Fig. S1).

Within the melanoma samples the *BRAF* expression levels varied between tumors harboring *BRAF* mutations and those wild-type for *BRAF* (*p* = 0.021; Fig. 1b). No difference in *BRAF* expression between *NRAS* mutated and *NRAS* wild-type was recorded (*p* > 0.1). Further, *BRAF* expression was higher in tumors with *BRAF* copy-number gains compared to those exhibiting a normal *BRAF* copy number (*p* = 0.028; Fig. 1c). Even though a low number of observations limits the strength of this analysis, comparing *BRAF* mRNA levels among tumors stratified for both *BRAF* mutation and copy-number status indicated that these two factors have an additive effect on *BRAF* expression (*p* = 0.005). Excluding tumors harboring *BRAF* copy-number gains from the analysis, *BRAF* expression levels remained higher among tumors harboring *BRAF* V600 mutations as compared to *BRAF* wild-type tumors (*p* = 0.014), suggesting an elevation of *BRAF* expression levels in tumors harboring the V600 mutations without additional gene copies in concert.

### Low *BRAF* and *NRAS* expression levels but not *BRAF* or *NRAS* mutation status are associated with clinical benefit from DTIC chemotherapy

Only 4 out of a total of 75 tumors evaluated for response to DTIC treatment revealed an objective response. To determine potential correlations between *BRAF* (and, subsequently, *NRAS*) alterations and outcome, we therefore compared the *BRAF* status among patients having an objective response or stable disease recorded 3 months after commencing treatment (defined here as clinical benefit; *n* = 15) versus patients with PD (*n* = 60) within the same time interval [23]. No correlations between *BRAF* copy number (number compared = 45) or mutation status (number compared = 75) and response to chemotherapy were recorded (*p* > 0.1 for both).

Similar to what was observed for *BRAF* mutations, *NRAS* mutation status was not associated with clinical outcome after 3 months on therapy (number compared = 75; *p* > 0.5). Neither did we observe any difference in response to therapy between patients with tumors harboring either a *BRAF* or an *NRAS* mutation on the one side and those wild-type for both genes on the other (number compared = 75; *p* > 0.5).

**Fig. 2:**
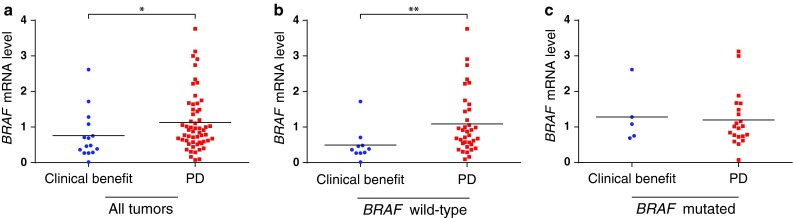
*BRAF* mRNA expression and response to DTIC treatment. *BRAF* mRNA expression among patients experiencing a clinical benefit or progressive disease (PD) 3 months after commencement of DTIC chemotherapy. **a** *BRAF* expression in the total patient group, **b** data as presented in **a**, but limited to patients with tumors harboring wild-type *BRAF*, **c** similar analysis performed for tumors harboring *BRAF* V600 mutations. *Asterisks* denote significance levels: *p ≤ 0.05, **p ≤ 0.005

In contrast, patients experiencing a clinical benefit to therapy after 3 months had lower *BRAF* mRNA expression levels compared to patients with PD (number compared = 72; *p* = 0.037; Fig. 2a). Limiting the analysis to patients with tumors expressing wild-type *BRAF* strengthened the association (number compared = 46; *p* = 0.005; Fig. 2b). The above comparisons were performed using rank tests, regarding *BRAF* expression level as a continuous variable. Assessing the robustness of the observed associations, we categorized *BRAF* expression as above or below the median, in the first quartile or in the first decile and compared the number of patients benefitting from therapy above or below these three cut-offs. For each analysis, we found low *BRAF* levels to be associated with clinical benefit to DTIC treatment in the total patient cohort as well as in the subgroup of patient with tumors wild-type for *BRAF* (*p* < 0.05 for all comparisons). In contrast, no association between *BRAF* expression level and benefit to therapy was observed among patients with tumors harboring *BRAF* mutations (number compared = 26; *p* > 0.5; Fig. 2c).

Similar to what was recorded for full-length *BRAF*, low levels of BRAFdel14–15 were associated with a clinical benefit 3 months following commencement of DTIC therapy (number compared = 67; *p* = 0.045; Fig. S2a). In contrast, no correlation between BRAFdel12–15 levels and response to DTIC chemotherapy were observed (number compared = 67; Fig. S2b).

**Fig. 3:**
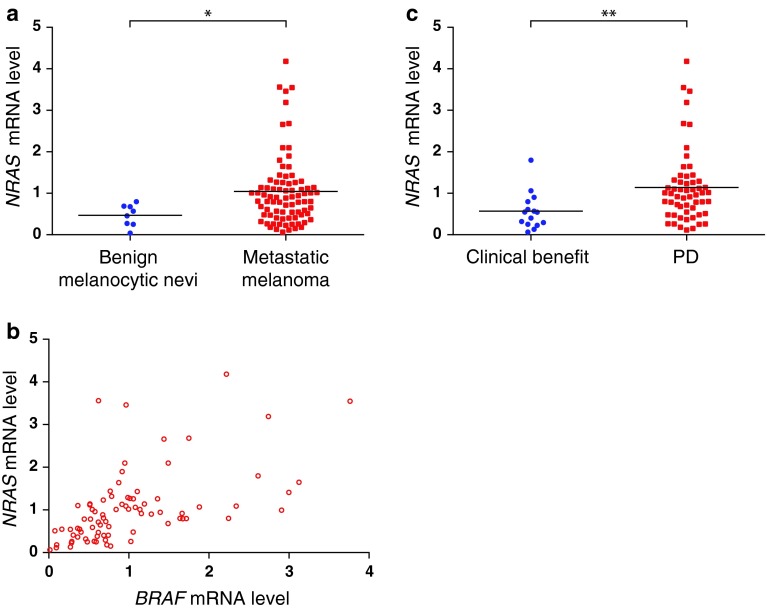
*NRAS* mRNA expression. **a** Expression levels of *NRAS* mRNA in benign melanocytic nevi (*n* = 8) and metastatic melanoma (*n* = 82). **b** Correlation plot showing the expression of *NRAS* in each tumor in relation to *BRAF* expression. **c** Expression levels of *NRAS* mRNA in patients experiencing a clinical benefit or progressive disease (PD) 3 months after commencement of chemotherapy. *Asterisks* denote significance levels: *p ≤ 0.05, **p ≤ 0.005

Based on the finding that *BRAF* expression levels were correlated to treatment response, we extended our analyses and determined *NRAS* mRNA levels in the same samples. Similar to what was seen for *BRAF*, *NRAS* expression levels were found to be significantly higher in malignant melanomas than in non-malignant nevi (*p* = 0.018; Fig. 3a). Moreover, there was a strong correlation between tumor *NRAS* and *BRAF* mRNA expression levels (*r*_s_ = 0.627; *p* < 0.001; Fig. 3b). While 33 tumors revealed *BRAF* as well as *NRAS* levels both above median values, and 33 tumors had both values below median, only 16 tumors had one gene expressed above and the other expressed below median level. Further, we found that, similar to *BRAF* expression, low levels of *NRAS* expression were associated with a clinical benefit following DTIC treatment (number compared = 72; *p* = 0.003; Fig. 3c). This correlation was, however, not strengthened by restricting the analysis to patients with tumors wild-type for *NRAS* (number compared = 55; *p* > 0.05).

### Low *BRAF* and *NRAS* expression is associated with overall and progression-free survival

Patients with clinical benefit from therapy revealed an improved progression-free and overall survival as compared to patients progressing at 3 months on therapy (number compared = 75; *p* < 0.001 for both; data not shown).

Next, we correlated progression-free and overall survival to *BRAF* and *NRAS* mutation status as well as *BRAF* and *NRAS* expression levels recorded as being below or above the median value.

**Fig. 4:**
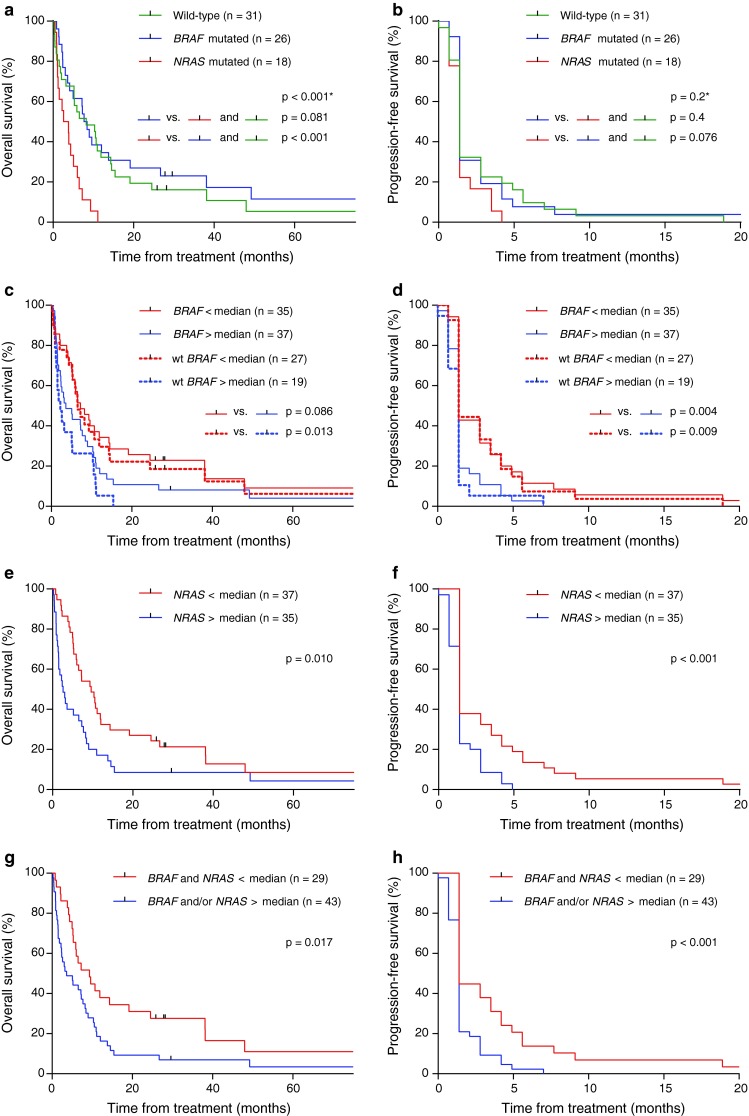
Progression-free and overall survival with respect to *BRAF*/*NRAS* status. Kaplan–Meier curves showing overall and progression-free survival: **a**, **b** patients with tumors harboring *BRAF* mutations or *NRAS* mutations, **c**, **d** effect of *BRAF* expression levels above or below the median value among patients evaluable for treatment response (median value determined among all patients). *Solid curves* represent all patients; *dashed curves* represent only patients with tumors harboring wild-type *BRAF*; **e**, **f** effect of *NRAS* expression levels above or below the median value among patients evaluable for treatment response (median value determined among all patients), **g**, **h** effect of combined *BRAF*/*NRAS* expression levels: one or both above compared to both below the median values. *All three groups included in the analysis

*BRAF* mutation status was not associated with either overall or progression-free survival (number compared = 75; Fig. 4a, b). Conversely, while no correlation between *NRAS* mutation status and progression-free survival was recorded, patients harboring *NRAS* mutations had a significantly shorter overall survival as compared to patients with tumors wild-type for *NRAS* (median survival 3.2 and 8.2 months, respectively; *p* < 0.001; Fig. 4a).

One potential confounding factor in assessing *BRAF* mutations as a prognostic factor could be lack of *BRAF* mutations in the visceral lesions, as well as a low prevalence of *BRAF* mutations in patients with brain metastases at inclusion, in as much as these patients may be expected to have a particularly poor prognosis. However, no effect of *BRAF* mutation status on survival was recorded after excluding patients with visceral biopsies or brain metastases from the analysis.

Analyzing patients with tumors mutated or wild-type for *BRAF* together (number compared = 72; Fig. 4c, d), *BRAF* expression below the median was associated with longer progression-free survival (*p* = 0.004). Excluding patients with tumors harboring V600 mutations from the analysis extended this association (number compared = 48; Fig. 4c, d); among patients with tumors wild-type for *BRAF*, low *BRAF* mRNA expression was associated with both a longer progression-free (*p* = 0.009) as well as an improved overall survival (median overall survival 6.5 months for low vs. 2.2 months for high *BRAF* mRNA expression; *p* = 0.013). In contrast, *BRAF* mRNA expression levels were not associated with overall or progression-free survival among patients with *BRAF* mutations (Fig. S3a, b). Comparing overall and progression-free survival among patients according to *NRAS* expression levels (number compared = 72; Fig. 4e, f) revealed expression levels below median value to be associated with an improved outcome in terms of progression-free (*p* < 0.001), as well as overall survival (median overall survival 9.6 months for low vs. 2.6 months for high *NRAS* expression; *p* = 0.01). Excluding patients with *NRAS* mutations did not strengthen this association (data not shown).

Combining *BRAF* and *NRAS* expression characteristics showed that patients with tumors expressing both genes below the median displayed an improved progression-free (*p* < 0.001) as well as overall (*p* = 0.017) survival as compared to patients with tumors in which either *BRAF* or *NRAS* or both were expressed above the median value (number compared = 72; Fig. 4g, h).

To assess the independent value of mutation status and expression of *BRAF* and *NRAS*, multivariate analysis was carried out using Cox regression entering serum LDH levels (available for 78 patients) and localization of most unfavorable metastasis present at treatment start (specified as locoregional, visceral or central nervous system metastases) together with *BRAF* and/or *NRAS* mutation/expression levels. Regarding progression-free survival, in general either *NRAS* expression levels or (when *BRAF* was added to the model) *BRAF* and *NRAS* expression levels combined (one or both elevated above median value) predicted a shorter time to progression (*p* < 0.05); neither serum LDH levels nor metastatic location were of significance. As for overall survival, both serum LDH and metastatic location consistently predicted survival (*p* values for both <0.005) together with either *NRAS* mutation status (*p* < 0.05) or *NRAS*/*BRAF* expression levels combined (*p* < 0.05).

## Discussion

The RAF group of proto-oncogenes consists of three family members (*A*-, *B*- and *CRAF*). Among these, *BRAF* has been shown to play a key role through activating mutations in malignant melanomas. BRAF and NRAS proteins are both subject to activating mutations; with V600E (BRAF) and Q61K/R/L (NRAS) being the most frequent ones [7, 8]. Our detection of *BRAF* mutations in 41 % of patients with cutaneous primary melanomas but lack of mutations among uveal- and mucosa-derived tumors are in accordance with findings by others [24, 26–29]. Furthermore, our finding of *NRAS* mutations in 20 % of all tumors is consistent with previous reports [29]. In contrast to others reporting the *BRAF* V600K mutation to occur in about 6–20 % of *BRAF*-mutated melanomas [30], we only observed this mutation in one out of 30 metastases (3.3 %). Regarding the nature of *BRAF* mutations, in contrast to V600E, the V600K mutation has been reported to be associated with cumulative sun-induced damage (CSD) [31]. As CSD can be assumed to be relatively uncommon in Norway as compared to most other geographical areas, this could explain the low prevalence of *BRAF* V600K mutations observed in the current study.

While our finding of few *BRAF* mutations in visceral metastases from cutaneous melanomas contrasts the finding of others [32], due to a limited number of observation this discrepancy may be caused by chance only.

The association between *BRAF* mutation status and copy-number gains has been reported by others as well [33–35]. To the best of our knowledge, this study is the first to report elevated *BRAF* expression levels in human melanomas harboring *BRAF* mutations without elevated gene copy number. Notably, this finding contrasts data from cell lines [15].

In the current study, two products of alternative splicing of the *BRAF* pre-mRNA were observed, BRAFdel14–15 and BRAFdel12–15, of which the former has previously been reported [18]. While others have described *BRAF* splice variants promoting oncogenic activity [19], each of the alternative splices detected in the current study lacks the catalytic domain considered crucial to RAF activation [36], suggesting these splices not to be oncogenic.

As has been observed previously in the case of *BRAF* [9], we found no correlation between *BRAF* or *NRAS* mutations and response to DTIC treatment in our patients. In contrast, a low *BRAF* mRNA expression level was associated with benefit from DTIC treatment. Notably, this association was particularly strong in patients with tumors wild-type for *BRAF*. These findings are somewhat contradictory. The *BRAF* V600E mutation has been reported to enhance enzyme activity in vitro by a factor of about 500 [11]; thus, we may envision a poor drug response as well as a poor prognosis for tumors harboring the activating V600E mutation as well. However, the biological role of BRAF may be different between tumors with wild-type or V600E-mutated BRAF. In tumors harboring the V600E mutation, BRAF seems to be the key proliferation driver [37]; thus, vemurafenib causes dramatic tumor shrinkage in patients with V600E-mutated tumors but not in tumors wild-type for BRAF [38]. Notably, *BRAF*-mutated tumors in general reveal gene expression profiles different from those harboring wild-type *BRAF* [39], and V600E-mutated BRAF has been shown to interact with CRAF in a manner different from wild-type BRAF [40]. Taken together, these findings indicate effects of V600E-mutated BRAF protein not to be related to wild-type BRAF “dosing activity” only. These observations may have clinical implications; while vemurafenib administered as monotherapy is ineffective in *BRAF* wild-type tumors where it may even activate RAF signaling [41], future drugs suppressing *BRAF* levels may potentially sensitize wild-type tumors to chemotherapy if administered in concert.

Our finding that low *NRAS* expression levels was associated with improved treatment outcome indirectly supports the hypothesis that low *BRAF* activity is associated with improved treatment response. Moreover, this underlines the observation that activation of the RAS/RAF kinase pathway by increased expression levels may cause biological effects different from that of activation by mutations. Interestingly, the mRNA expression of *BRAF* and *NRAS* were positively correlated, in contrast to the mutation distribution of these genes, which were mutually exclusive.

Conflicting evidence has reported *BRAF* and *NRAS* mutations to be associated with a poor prognosis in melanoma [4–6, 9, 10, 42, 43]. Considering unresectable stages III and IV disease, our data are consistent with Jakob et al. [10] who reported shorter survival among patients with tumors harboring *NRAS* mutations. While our results do not support *BRAF* mutations to be associated with a poor prognosis in advanced melanoma, as suggested by Long et al. [9], their study reported survival for patients with *BRAF*-mutated tumors found ineligible for BRAF inhibitor trials who, as stated by the authors, are expected to be a poor-survival group due to intercurrent medical problems [9].

While the data currently presented should be interpreted carefully due to the limited number of patients studied, the association of low *BRAF* and *NRAS* expression with benefit from anti-tumor therapy as well as improved relapse-free and overall survival is interesting. Although the multivariate analyses carried out suggested an independent association between *BRAF*/*NRAS* expression levels and outcome, the results should be interpreted carefully due to the limited number of observations. While these findings may suggest that low *BRAF*/*NRAS* expression levels could be both a predictive as well as a prognostic factor [44], validation in independent studies are needed. For some patients, stable disease after 3 months on therapy could reflect slow tumor growth and may not necessarily signal response to DTIC therapy. As such, the effects of *NRAS*/*BRAF* status on survival may reflect an enhanced effect of treatment or, alternatively, tumor biology independent of drug therapy [44].

In conclusion, we present data linking *BRAF*, as well as *NRAS*, mRNA expression levels to outcome in advanced melanoma. Our data further indicate that low *BRAF* and *NRAS* expression levels may predict for benefit to DTIC chemotherapy. For patients with tumors harboring wild-type *BRAF* treated with DTIC chemotherapy, a low level of *BRAF* expression was associated with longer overall and progression-free survival, whereas low *NRAS* expression was associated with improved progression-free and overall survival irrespective of mutation status. Further studies are warranted to confirm a potential predictive and prognostic role of *BRAF* and *NRAS* expression levels in advanced melanomas.

## Electronic supplementary material

Below is the link to the electronic supplementary material.

Supplementary material 1 (PDF 853 kb)
