# Supplementary material for: Low BRAF and NRAS expression levels are associated with clinical benefit from DTIC therapy and prognosis in metastatic melanoma
Source: Clin Exp Metastasis. 2013 May 15;30(7):867–76. doi: 10.1007/s10585-013-9587-4 (PMC3837233; doi:10.1007/s10585-013-9587-4)
Supplement: Supplementary file 1 — Supplementary material 1 (PDF 853 kb) [file 10585_2013_9587_MOESM1_ESM.pdf]

Code in study	Age at treatment start	Sex	Disease stage at inclusion	Location of primary	Location of biopsy	NRAS codon 61	BRAF codon 600	BRAF copy-number	BRAF expression relative to RPLP2	NRAS expression relative to RPLP2	Response to DTIC after 3 months
MM 01	62	M	IV	Truncus	Axillary lymph node	wt	wt	Unknown	1.439	2.66	PD
MM 02	59	F	IV	Lower extremities	Subcutaneous truncus	C181A (Q61K)	wt	Unknown	0.361	0.36	PD
MM 03	53	M	IV	Head	Subcutaneous	wt	wt	Normal	0.576	0.96	PD
MM 04	61	F	IV	Lower extremities	No information	A182G (Q61R)	wt	Normal	0.997	1.02	PD
MM 05	46	M	IV	Lower extremities	No information	wt	wt	Unknown	0.386	0.55	PR
MM 06	49	M	IV	Lower extremities	Liver	A182G (Q61R)	wt	Normal	2.220	4.18	PD
MM 08	68	M	IV	No primary	Subcutaneous truncus	C181A, A182G (Q61R)	wt	Normal	0.482	0.25	SD
MM 09	38	M	IV	Acral	Subcutaneous	wt	wt	Normal	0.270	0.13	CR
MM 10	65	F	IV	Truncus	Subcutaneous truncus	wt	wt	Normal	1.194	1.14	PD
MM 11	52	M	IV	No primary	Axillary lymph node	wt	wt	Normal	0.288	0.26	PD
MM 12	86	M	IV	Head	Subcutaneous truncus	wt	G1798A, T1799A (V600K)	Normal	0.076	0.51	PD
MM 13	51	M	IV	Truncus	Subcutaneous truncus	A182G (Q61R)	wt	Normal	0.364	1.10	PD
MM 14	59	F	IV	Upper extremities	Subcutaneous truncus	A182G (Q61R)	wt	Normal	0.915	1.13	PD
MM 15	75	M	IV	Acral	Liver	wt	wt	Unknown	2.909	0.99	PD
MM 16	36	M	III	No primary	Axillary lymph node	wt	T1799A (V600E)	Unknown	0.689	0.40	PR
MM 17	66	F	IV	Upper extremities	Subcutaneous truncus	A182T (Q61L)	wt	Unknown	0.873	1.64	PD
MM 18	53	F	IV	Lower extremities	Subcutaneous truncus	wt	wt	Normal	0.681	1.23	PD
MM 19	32	F	IV	Truncus	Subcutaneous truncus	wt	T1799A (V600E)	Increased	2.614	1.80	SD
MM 20	51	M	IV	Lower extremities	Subcutaneous lower extremities	A182T (Q61L)	wt	Normal	0.402	0.48	PD
MM 21	63	M	IV	No primary	Subcutaneous truncus	A182G (Q61R)	wt	Normal	0.966	3.46	PD

MM 22	71	M	IV	Upper extremities	Subcutaneous truncus	wt	T1799A (V600E)	Increased	1.882	1.07	PD
MM 23	60	F	IV	GI-tractus	Liver	wt	wt	Unknown	3.763	3.55	PD
MM 24	63	F	IV	Truncus	Axillary lymph node	wt	T1799A (V600E)	Normal	1.676	0.79	PD
MM 25	61	F	IV	Lower extremities	Subcutaneous truncus	wt	T1799A (V600E)	Increased	2.999	1.41	PD
MM 27	80	M	IV	Truncus	Subcutaneous truncus	C181A (Q61K)	wt	Normal	0.918	1.90	PD
MM 29	68	M	IV	Eye	Liver	wt	wt	Unknown	0.988	1.29	PD
MM 30	n/a[**]	F	IV	Lower extremities	Inguinal lymph node	wt	T1799A (V600E)	Increased	0.951	2.10	n/a[**]
MM 31	57	M	IV	No primary	Subcutaneous truncus	wt	wt	Increased	2.742	3.19	PD
MM 32	75	F	IV	Head	Abdominal lymph node	A182G (Q61R)	wt	Unknown	1.749	2.68	PD
MM 33	70	M	IV	Truncus	Spleen	wt	wt	Unknown	n/a[*]	n/a[*]	PD
MM 34	76	F	IV	Head	Subcutaneous lymph node	wt	wt	Normal	1.492	2.10	PD
MM 35	74	M	IV	No primary	Liver	wt	wt	Unknown	0.674	0.89	PD
MM 36	n/a[‡]	M	IV	Eye	Liver	wt	wt	Unknown	0.683	0.81	n/a[‡]
MM 37	70	M	IV	Truncus	Cervical lymph node	wt	T1799A (V600E)	Normal	0.786	1.32	PD
MM 38	49	M	IV	Truncus	Subcutaneous truncus	wt	T1799A (V600E)	Increased	0.743	0.41	PD
MM 39	n/a[§]	F	IV	Eye	Liver	wt	wt	Unknown	1.393	0.94	n/a[§]
MM 40	33	M	IV	No primary	Cervical lymph node	wt	T1799A (V600E)	Normal	0.619	0.47	PD
MM 41	n/a[§]	F	IV	No primary	Subcutaneous truncus	wt	T1799A (V600E)	Normal	1.139	1.01	n/a[§]
MM 42	74	M	IV	Truncus	Subcutaneous upper extremity	wt	wt	Normal	0.768	1.44	PD
MM 43	31	F	IV	Truncus	Subcutaneous upper extremity	wt	T1799A (V600E)	Increased	1.080	1.06	SD
MM 44	43	F	IV	No primary	Subcutaneous truncus	wt	T1799A (V600E)	Normal	0.934	1.09	PD
MM 45	n/a[ǁ]	F	III	Truncus	Pelvic lymph node	wt	T1799A (V600E)	Increased	1.058	1.26	n/a[ǁ]

MM 47	61	M	IV	No primary	Subcutaneous upper extremity	C181A (Q61K)	wt	Normal	0.283	0.23	SD
MM 48	56	F	IV	Truncus	Liver	wt	wt	Unknown	0.513	1.12	PD
MM 49	45	M	IV	Head	Liver	wt	wt	Unknown	0.707	0.29	SD
MM 50	73	M	IV	Two primaries; 1 Skin, 2 Eye	Subcutaneous truncus	wt	wt	Normal	0.269	0.54	SD
MM 51	55	M	IV	Truncus	Subcutaneous truncus	wt	wt	Increased	0.568	0.27	PD
MM 52	68	F	IV	No primary	Subcutaneous truncus	wt	T1799A (V600E)	Normal	0.522	0.78	PD
MM 53	68	F	IV	Truncus	Subcutaneous head	wt	T1799A (V600E)	Increased	1.106	1.43	PD
MM 54	43	F	IV	Eye	Liver	C181T (Q61Ter)[∫]	wt	Unknown	2.246	0.80	PD
MM 55	36	F	IV	No primary	Cervical lymph node	wt	T1799A (V600E)	Normal	1.489	0.68	PD
MM 56	54	M	IV	No primary	Abdominal lymph node	wt	T1799A (V600E)	Unknown	1.025	0.26	PD
MM 57	79	F	IV	Truncus	Subcutaneous upper extremity	C181A (Q61K)	wt	Normal	0.620	0.71	PD
MM 58	68	M	IV	Head	Subcutaneous neck	wt	T1799A (V600E)	Normal	1.014	1.27	PD
MM 59	64	F	IV	Genital	Abdominal lymph node	wt	wt	Unknown	0.305	0.41	PD
MM 60 A	51	F	IV	Mucosa	Liver	wt	wt	Unknown	0.543	1.01	PD
MM 60 M	n/a[‡]	F	IV	Truncus	Axillary lymph node	wt	T1799A (V600E)	Normal	0.514	1.14	n/a[‡]
MM 61	67	M	IV	Truncus	Subcutaneous head	wt	T1799A (V600E)	Normal	1.157	0.92	PD
MM 62	51	F	IV	Truncus	Subcutaneous head	C181A (Q61K)	wt	Unknown	0.014	0.07	SD
MM 64	n/a[‡]	F	IV	Upper extremities	Subcutaneous truncus	wt	wt	Increased	0.100	0.18	n/a[‡]
MM 65	67	F	IV	Acral	Liver	wt	wt	Unknown	1.638	0.80	PD
MM 66	50	M	IV	Truncus	Axillary lymph node	A182G (Q61R)	wt	Unknown	0.644	0.65	PD
MM 67	56	M	IV	GI-tractus	Liver	wt	wt	Unknown	0.094	0.12	PD
MM 68	72	M	IV	Truncus	Supraclavicular lymph node	wt	wt	Normal	0.460	0.32	SD

MM 69	68	F	IV	GI-tractus	Liver	wt	wt	Unknown	0.443	0.79	PD
MM 71	84	M	IV	Truncus	Axillary lymph node	wt	wt	Normal	0.712	0.73	PD
MM 72	59	M	IV	No primary	Liver	A182G (Q61R)	wt	Unknown	n/a[*]	n/a[*]	PD
MM 73	n/a[||]	M	IV	Upper extremities	Subcutaneous upper extremity	C181A (Q61K)	wt	Normal	0.621	3.56	n/a[||]
MM 76	58	M	IV	Truncus	Liver	wt	wt	Unknown	2.341	1.09	PD
MM 77	63	M	IV	Truncus	Supraclavicular lymph node	wt	wt	Unknown	1.718	0.80	SD
MM 78	n/a[||]	M	III	Truncus	Axillary lymph node	wt	wt	Normal	0.605	0.38	n/a[||]
MM 79	74	M	IV	Head	Subcutaneous truncus	C181A (Q61K)	wt	Unknown	1.055	0.48	PD
MM 80	80	F	IV	Truncus	Subcutaneous truncus	wt	wt	Normal	0.163	0.55	PD
MM 81	n/a[§]	F	IV	Upper extremities	Subcutaneous upper extremity	wt	wt	Normal	0.546	0.59	n/a[§]
MM 82	76	M	IV	Lower extremities	Axillary lymph node	wt	T1799A (V600E)	Normal	1.665	0.92	PD
MM 83	75	F	IV	Lower extremities	Subcutaneous truncus	wt	T1799A (V600E)	Increased	1.281	0.90	SD
MM 84	60	M	IV	No primary	Kidney	wt	wt	Unknown	n/a[*]	n/a[*]	PD
MM 85	68	M	IV	Truncus	Liver	wt	T1799A (V600E)	Unknown	0.593	0.25	PD
MM 86	61	M	IV	Truncus	Axillary lymph node	wt	T1799A (V600E)	Normal	0.751	0.61	SD
MM 87	74	M	IV	Truncus	Liver	wt	T1799A (V600E)	Unknown	0.773	0.15	PD
MM 88	74	F	IV	Acral	Subcutaneous lower extremity	wt	wt	Normal	0.360	0.57	SD
MM 90	56	F	IV	Truncus	Liver	wt	T1799A (V600E)	Unknown	0.725	0.18	PD
MM 93	76	M	IV	Truncus	Axillary lymph node	wt	T1799A (V600E)	Normal	1.359	1.26	PD
MM 94	25	F	IV	Lower extremities	Inguinal lymph node	wt	T1799A (V600E)	Normal	3.125	1.65	PD
MM 95	75	M	IV	Truncus	Axillary lymph node	wt	T1799A (V600E)	Normal	0.841	1.01	PD

[*] Expression not evaluated due to lack of material

[**] Declined treatment because of high age
[‡] Rapidly deteriorating condition before commencing chemotherapy
[∫] This novel mutation was analysed as all other *NRAS* mutation, although it does not appear to be activating. Leaving this sample out of analyses did not alter the associations found in the study.

[§] Withdrew from treatment
[||] Tissue samples taken in conjunction with therapeutic surgery for first relapse; the patient was considered candidate for DTIC treatment in case of a second relapse.

Supplemetary table S2: PCR primers and conditions

Target gene	Nested PCR reaction	Primer	Annealing temperature
BRAF (cDNA)	1st	Forward: ACTTGGTAGACGGGACTC<br>Reverse: CTCAATAGAGGCGAGAATTT	56 °C
	2nd	Forward: TTGGATCTGGATCATTTGGAACA<br>Reverse: CTCAATAGAGGCGAGAATTT	56 °C
BRAF (genomic)		Forward: TCATAATGCTTGCTCTGATAGGA<br>Reverse: GGCCAAAAATTTAATCAGTGGA	56 °C
NRAS (cDNA)	1st	Forward: GTCTGTGGTCCTAAATCTGTCC<br>Reverse: GTGAGACTGAAGACAGCAACAG	50 °C
	2nd	Forward: GCAGTGGAGCTTGAGGTTC<br>Reverse: GGTGTCAGTGCAGCTTGAA	50 °C
BRAF (full-length)		Forward: CCATTTTGTGGATGGCACCA<br>Reverse: CTCTTCATGGCTTTTGGACAG<br>Probe: 6FAM-ACGAGGATACCTGTCTCCAGATCTCAGTA-BBQ	64 °C
BRAF (del14-15)		Forward: CAGGGCATGGAGCACC<br>Reverse: CTCTTCATGGCTTTTGGACAG<br>Probe: 6FAM-ACGAGGATACCTGTCTCCAGATCTCAGTA-BBQ	62 °C
BRAF (del12-15)		Forward: GAAAGTGGCATGGCACCA<br>Reverse: TTTGAGGCACTCTGCCATTA<br>Probe: 6FAM- TGGTGGGACGAGGATACCTGTCTCC-BBQ	62 °C
NRAS		Forward: TGCCATGTGTGGTGATGTAA<br>Reverse: GCTTCTCTGTGAGACTGAAGAC<br>Probe: 6FAM- AGGGTGTCAGTGCAGCTTGAAAGT-BBQ	58 °C
RPLP2		Forward: GACCGGCTCAACAAGGTTAT<br>Reverse: CCCCACCAGCAGGTACAC<br>Probe: Cy5- AGCTGAATGGAAAAAACATTGAAGACGTC-BBQ	53 °C

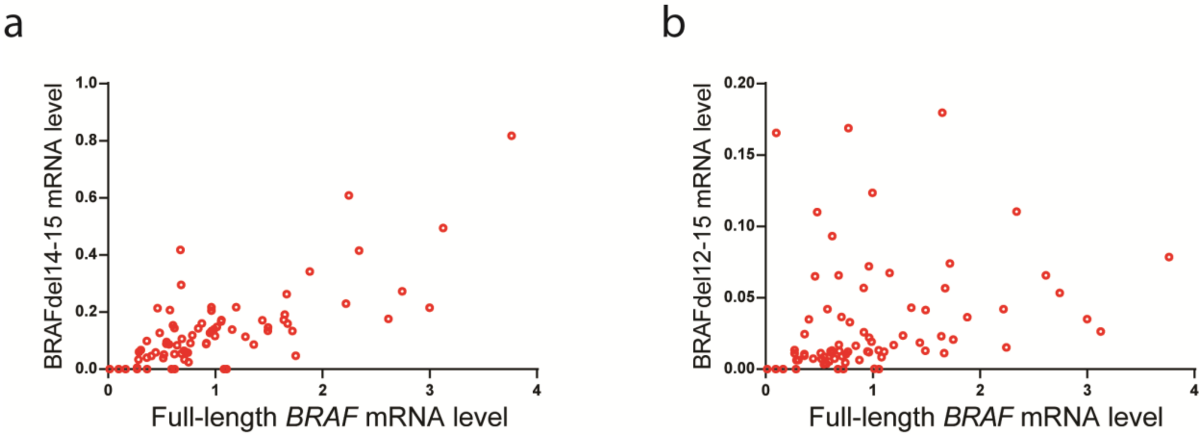

Supplemtary figure S1: Correlation plots showing the expression of (a) BRAFdel14-15, and (b) BRAFdel12-15 in relation to full-length *BRAF* mRNA expression.

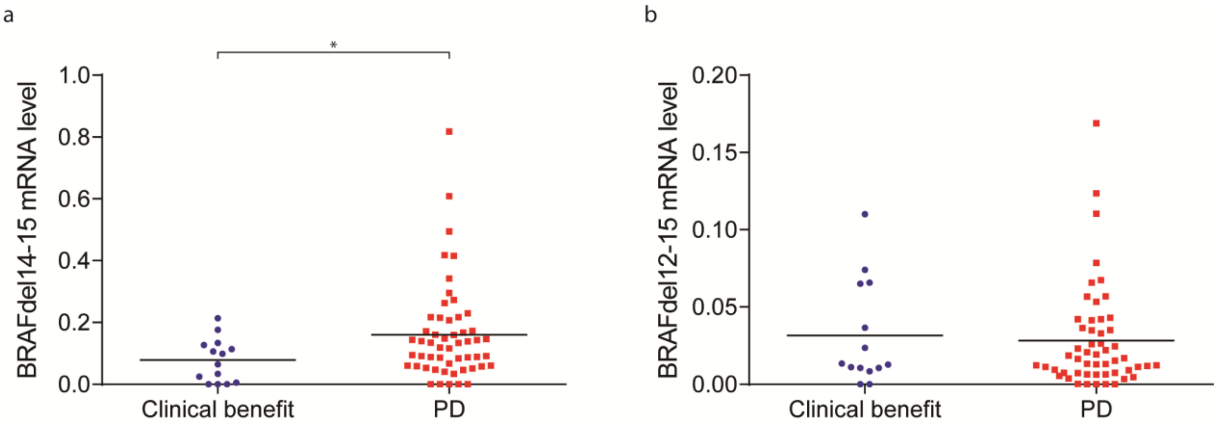

Supplementary figure S2: Expression of *BRAF* splice variants BRAFdel14-15 (a) and BRAFdel12-15 (b) among patients experiencing a clinical benefit or with disease progression 3 months following chemotherapy commencement.

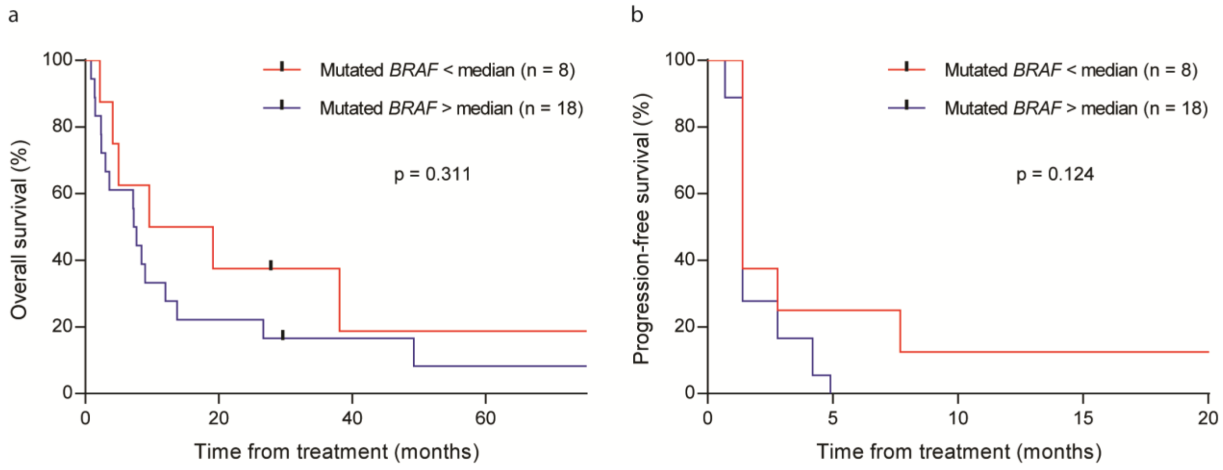

Supplementary figure S3: Overall and progression-free survival among patients with mutated *BRAF* according to *BRAF* mRNA expression.
